# A Paternal Fish Oil Diet Preconception Reduces Lung Inflammation in a Toxicant-Driven Murine Model of New Bronchopulmonary Dysplasia

**DOI:** 10.3390/md21030161

**Published:** 2023-02-27

**Authors:** Jelonia T. Rumph, Victoria R. Stephens, Sharareh Ameli, LaKendria K. Brown, Kayla J. Rayford, Pius N. Nde, Kevin G. Osteen, Kaylon L. Bruner-Tran

**Affiliations:** 1 Women’s Reproductive Health Research Center, Department of Obstetrics and Gynecology, Vanderbilt University School of Medicine, Nashville, TN 37232, USA; 2 Department of Microbiology, Immunology and Physiology, Meharry Medical College, Nashville, TN 37208, USA; 3 Department of Pathology, Microbiology and Immunology, Vanderbilt University School of Medicine, Nashville, TN 37232, USA; 4 VA Tennessee Valley Healthcare System, Nashville, TN 37232, USA

**Keywords:** bronchopulmonary dysplasia, fish oil, toxicants, inflammation, neonatal, lung, dioxin, formula, therapeutics

## Abstract

New bronchopulmonary dysplasia (BPD) is a neonatal disease that is theorized to begin in utero and manifests as reduced alveolarization due to inflammation of the lung. Risk factors for new BPD in human infants include intrauterine growth restriction (IUGR), premature birth (PTB) and formula feeding. Using a mouse model, our group recently reported that a paternal history of 2,3,7,8-tetrachlorodibenzo-p-dioxin (TCDD) exposure increased his offspring’s risk of IUGR, PTB, and new BPD. Additionally, formula supplementation of these neonates worsened the severity of pulmonary disease. In a separate study, we reported that a paternal preconception fish oil diet prevented TCDD-driven IUGR and PTB. Not surprisingly, eliminating these two major risk factors for new BPD also significantly reduced development of neonatal lung disease. However, this prior study did not examine the potential mechanism for fish oil’s protective effect. Herein, we sought to determine whether a paternal preconception fish oil diet attenuated toxicant-associated lung inflammation, which is an important contributor to the pathogenesis of new BPD. Compared to offspring of standard diet TCDD-exposed males, offspring of TCDD-exposed males provided a fish oil diet prior to conception exhibited a significant reduction in pulmonary expression of multiple pro-inflammatory mediators (*Tlr4*, *Cxcr2*, *Il-1 alpha*). Additionally, neonatal lungs of pups born to fish oil treated fathers exhibited minimal hemorrhaging or edema. Currently, prevention of BPD is largely focused on maternal strategies to improve health (e.g., smoking cessation) or reduce risk of PTB (e.g., progesterone supplementation). Our studies in mice support a role for also targeting paternal factors to improve pregnancy outcomes and child health.

## 1. Introduction

New Bronchopulmonary Dysplasia (BPD) is a neonatal lung disease that is among the leading causes of death in premature infants. Epidemiology and animal models suggest that risk factors for this disease include intrauterine growth restriction (IUGR), premature birth (PTB), and formula feeding [1,2]. Chorioamnionitis and maternal smoking have each been linked to the development of new BPD in neonates [3,4,5] and supports the theory that this disease begins during pregnancy. TCDD (2,3,7,8-tetrachlorodibenzo-p-dioxin) is a common environmental contaminant present in cigarette smoke as well as car exhaust and forest fires. Our group previously reported that less than 50% of adult males with a history of in utero exposure to TCDD (F1_TCDD_ males) were fertile and that the placentae arising in their unexposed partners exhibited heightened inflammation compared to control pregnancies [6,7]. These pregnancies frequently resulted in spontaneous PTB as well as pups born small for their gestational age [7,8]. Since IUGR and PTB are well-known risk factors for new BPD, our group recently assessed the incidence of this disease in offspring of TCDD-exposed males (F2_TCDD_ mice). We demonstrated that new BPD was common in F2_TCDD_ pups, and that formula supplementation of these neonates heightened the severity of disease [9,10]. 

Relevant to the current study, we also previously reported that providing F1_TCDD_ males a fish oil supplemented diet prior to mating eliminated the risk of new BPD in F2_TCDD_ mice. These studies further demonstrated that a paternal fish oil diet preconception improved postnatal growth, lung alveolarization, and the risk of interalveolar red blood cell infiltration in F2 animals. Importantly, the presence of blood within the interalveolar space is a sign of pulmonary hypertension in human infants and is often a consequence of new BPD [9]. 

Human infants who develop new BPD are also susceptible to inflammation-driven hemorrhaging and edema [11,12]. Although our previous studies did not specifically explore lung inflammation in neonatal pups born to fish oil treated fathers, a number of investigators have demonstrated that direct fish oil supplementation reduces lung inflammation and edema in animal models [13,14]. Importantly, this treatment was not associated with increased risk of hemorrhage in either human infants or mice [14,15]. Finally, inflammation-driven fibrosis has been documented in numerous tissues [16] as well as in association with BPD [17]. Fish oil fatty acid supplementation was found to reduce collagen deposition in an animal model [18] while human supplementation was shown to protect against the development of interstitial lung disease [19]. There is also evidence that long-term supplementation with fish oil fatty acids may benefit patients with cystic fibrosis [20]. However, to our knowledge, the impact of the paternal preconception diet on neonatal lung disease has not previously been investigated.

In the present study, we evaluated the impact of both paternal and neonatal diet on lung inflammation and associated comorbidities in a TCDD-driven murine model of new BPD. We found that offspring of control males exhibited normal morphology regardless of the paternal diet; however, disease-associated changes were noted in the majority of F2_TCDD_ pups. Interestingly, neonatal formula supplementation had little impact on lung health in the absence of paternal toxicant exposure. We further found that a paternal fish oil diet preconception provided to F1_TCDD_ males attenuated pulmonary expression of pro-inflammatory cytokines in the F2 generation *regardless of the neonatal diet*. Reduced inflammation was associated with a reduction in lung hemorrhaging, edema, and fibrosis. Our findings demonstrate that intervening with a fish oil diet preconception reduces the risk of toxicant-associated new BPD in the F2 generation by attenuating lung inflammation and its sequelae. 

## 2. Results

### 2.1. A Paternal Fish Oil Diet Reduced the Risk of Lung Hemorrhaging and Edema in F2_TCDD_ Pups

**Figure 1:**
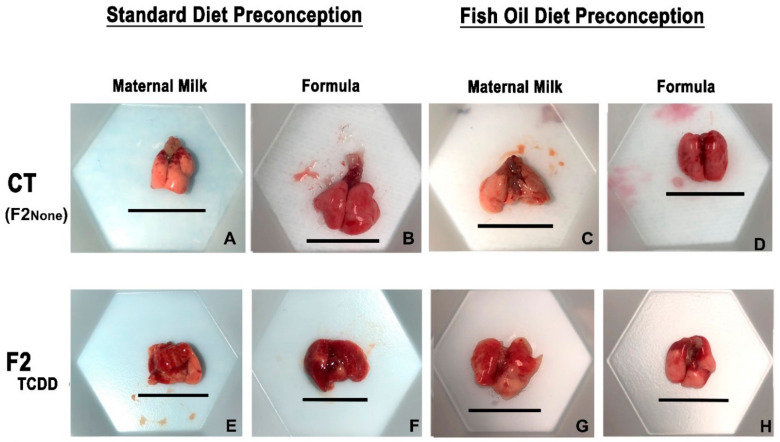
A paternal fish oil diet reduced toxicant-driven lung hemorrhaging. Representative images of gross lung anatomy of neonatal lungs on postnatal day 11 taken at 15× magnification. Scale bar = 11.5 mm.

Our first goal was to determine whether in utero TCDD exposure of fathers (F1_TCDD_ males) was associated with lung hemorrhaging and edema in their offspring (F2_TCDD_ pups) (Figure 1; The pup nomenclature used through the text is listed in Table 1). We also examined the impact of both the paternal and neonatal diet on these parameters. To assess these endpoints, all experimental groups were compared to offspring of unexposed controls (F2_None_ pups). 

F2_None_ and F2_None_/Form pups displayed healthy lungs with no visible signs of hemorrhaging (Figure 1B). The gross lung anatomy of F2_None_/Fish pups was also comparable to F2_None_ pups (Figure 1C) and was unaffected by formula supplementation (F2_None_/Fish/Form pups) (Figure 1D).

In contrast to F2_None_ offspring, abnormal gross anatomy of the lungs was observed in approximately 35% of F2_TCDD_ pups. Additionally, F2_TCDD_ pups also exhibited hemorrhaging, noted by the coagulation of blood on the surface of the lung (Figure 1E). Lung hemorrhaging was most severe among F2_TCDD_/Form pups and affected about 45% of these animals (Figure 1F). However, paternal fish oil supplementation improved these outcomes resulting in 100% of F2_TCDD_/Fish pups exhibiting normal lung anatomy with little-to-no hemorrhaging and was comparable to F2_None_ pups (Figure 1G). Minimal lung hemorrhaging was also noted in F2_TCDD_/Fish/Form pups (Figure 1H).

**Figure 2:**
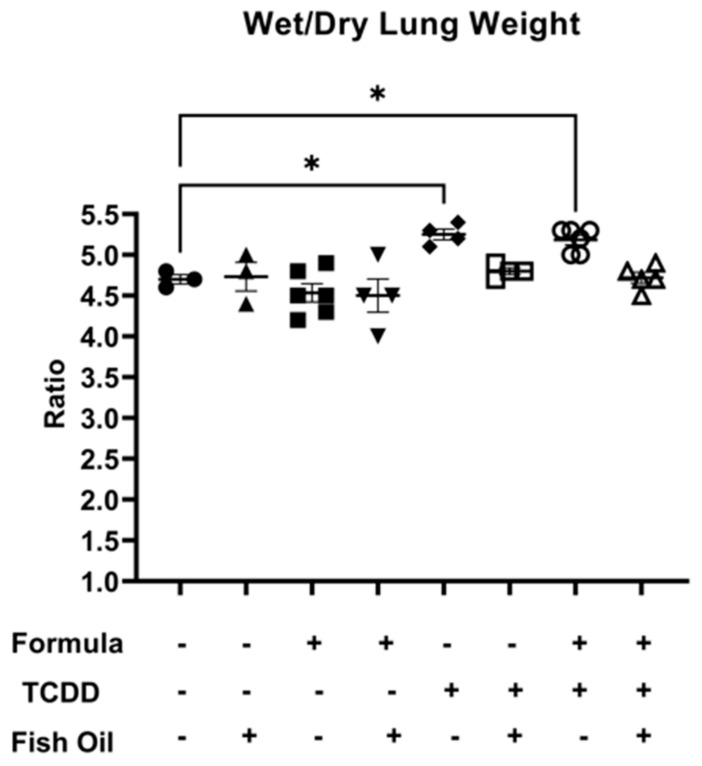
A paternal fish oil diet preconception reduced the risk of toxicant-driven edema in F2_TCDD_ pups. Average wet-to-dry lung weights (* *p* < 0.05).

Next, we measured lung edema in all groups by assessing the mean wet-to-dry lung weight ratio. Among all groups, there were no significant differences when considering wet lung weight or dry lung weight individually (Supplemental Figures S1 and S2). F2_None_ pups, independent of paternal and neonatal diet, did not exhibit lung edema as characterized by a wet-to-dry lung weight ratio of 5 or greater. However, F2_TCDD_ (*p* = 0.0168) and F2_TCDD_/Form (*p* = 0.0246) pups displayed a significant increase in wet-to-dry lung weight compared to F2_None_ pups. Additionally, 100% (4/4) of F2_TCDD_ pups exhibited a wet-to-dry lung weight ratio of 5 or greater. Interestingly, 60% (3/5) of F2_TCDD_/Form pups exhibited a wet-to-dry lung weight ratio of 5 or greater. None of the F2_TCDD_/Fish (*N* = 4) and F2_TCDD_/Fish/Form pups (*N* = 5) exhibited a wet-to-dry lung weight ratio of 5 or greater (Figure 2).

### 2.2. A Paternal Fish Oil Diet Preconception Reduced Pulmonary Expression of Toxicant and Formula Driven Pro-Inflammatory Mediators in F2_TCDD_ Pups

**Figure 3:**
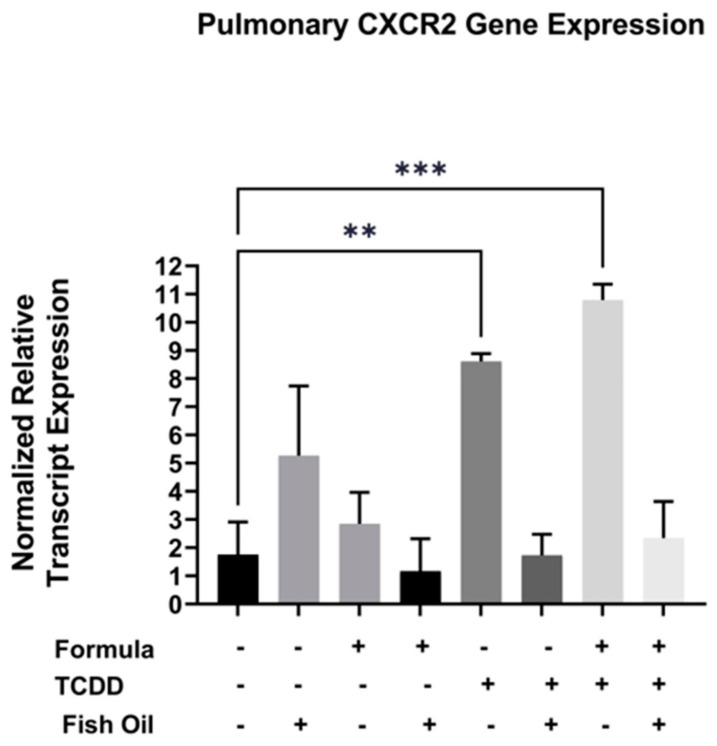
A paternal fish oil diet preconception reduced toxicant-driven pulmonary *Cxcr2* gene expression in F2_TCDD_ pups. Lung RNA was isolated from all groups and subjected to qRT-PCR to measure *Cxcr2* mRNA expression. For this set of experiments, each exposure and treatment group consisted of 3 pups from different litters. (** *p* < 0.01, *** *p* < 0.001).

C*xcr2*, a CXC chemokine receptor, is commonly overexpressed in pulmonary diseases such as acute lung disease, chronic obstructive pulmonary disease, and new BPD [21,22,23]. Additionally, TCDD exposure can elicit immune responses in a *Cxcr2*-dependent manner [24]. Herein, we evaluated whether a paternal history of TCDD exposure was associated with increased pulmonary expression of *Cxcr2* in neonatal offspring. We additionally assessed the impact of both the paternal diet (standard or fish oil) and neonatal diet (with or without formula supplementation) on this outcome**.** We measured the mRNA transcript level of *Cxcr2* in the lungs of all F2 neonates. Our studies revealed that F2_TCDD_ (*p* = 0.0007) and F2_TCDD_/Form pups (*p* = 0.0077) exhibited significant increases in the normalized relative transcript expression of *Cxcr2* when compared to F2_None_ pups. Intervening with a paternal fish oil diet preconception normalized the relative transcript expression of *Cxcr2* in F2_TCDD_/Fish and F2_TCDD_/Fish/Form pups to F2_None_ pups (Figure 3). It is interesting to note that formula, in the absence of paternal TCDD exposure, had little impact on pulmonary *Cxcr2* gene expression. In contrast, expression of this gene was highest in F2_TCDD_/Form pups suggesting a potential synergistic effect of paternal exposure and neonatal diet. 

**Figure 4:**
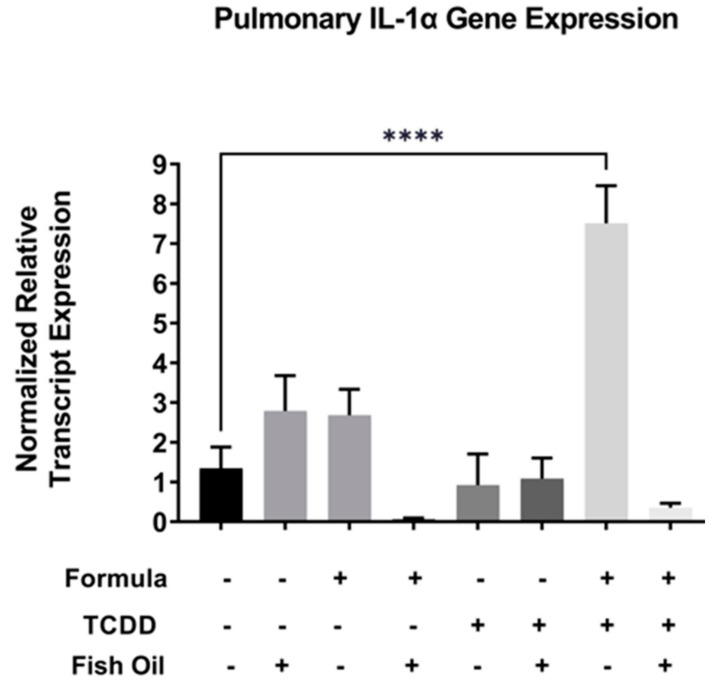
A paternal fish oil diet preconception reduced pulmonary gene expression of toxicant and formula-driven *Il-1 alpha* in F2_TCDD_ pups. Lung RNA was isolated from all groups and subjected to qRT-PCR for *Il-1 alpha*. For this set of experiments, each exposure and treatment group consisted of 3 pups from multiple litters (**** *p* < 0.0001).

Activation of the *Il-1* family of cytokines is downstream of *Cxcr2* activation and aberrant expression of the *Il-1* family has been implicated in pulmonary diseases such as new BPD [25,26]. Therefore, we assessed the relative mRNA transcript expression of *Il-1 alpha* in F2_None_ and F2_TCDD_ groups. We found that *Il-1 alpha* expression in lungs of F2 pups was not impacted by formula alone (F2_None_/Form) or paternal toxicant exposure alone (F2_TCDD_). In contrast, *Il-1 alpha* expression was significantly increased in F2_TCDD_/Form pups (*p* < 0.0001), strongly suggesting a synergistic effect of these treatments. Intervening with a paternal fish oil diet preconception reduced the relative expression level of *Il-1 alpha* among F2_TCDD_/Fish/Form pups when compared to F2_TCDD_/Form pups (Figure 4).

**Figure 5:**
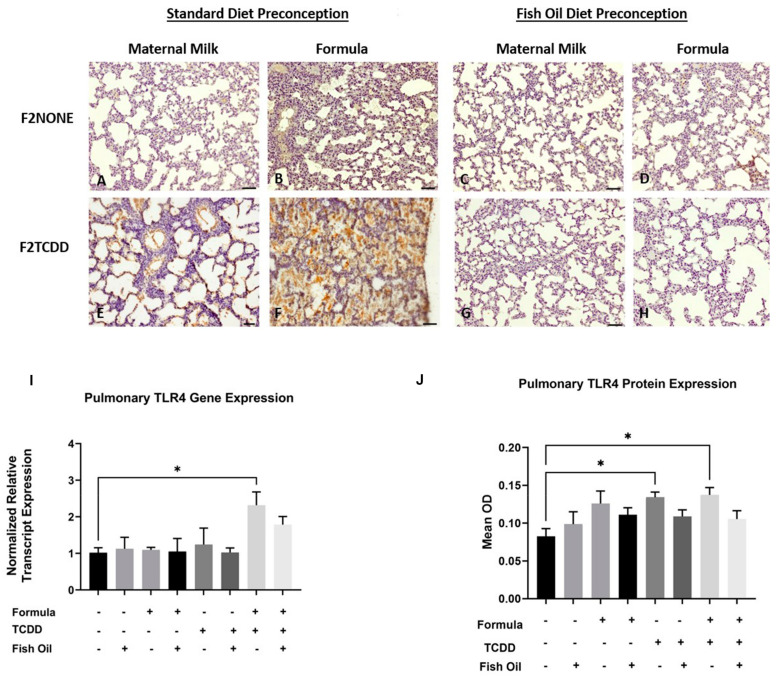
A paternal fish oil diet normalized pulmonary *Tlr4* gene and protein expression in F2_TCDD_ pups. Representative images from multiple lungs (*N* = 3 or more /group) of *Tlr4*-stained perfused lung tissue on postnatal day 11. Original magnification 200× (**A**–**H**); F2_NONE_ (**A**), F2_NONE_/Form (**B**), F2_NONE_/Fish (**C**), F2_NONE_/Fish/Form (**D**), F2_TCDD_ (**E**), F2_TCDD_/Form (**F**), F2_TCDD_/Fish (**G**), F2_TCDD_/Fish/Form (**H**). Lung RNA was isolated from all groups and subjected to qRT-PCR for *Tlr4* (**I**). TLR4 protein expression was determined by computer-assisted quantification of DAB substrate from lung immunohistochemistry experiments (**J**). For this set of experiments, each exposure and treatment group consisted of 3 pups from multiple litters (* *p* < 0.05).

We previously reported that, in addition to new BPD, F2_TCDD_ pups are susceptible to developing necrotizing enterocolitis (NEC) [9,10]. NEC is a life-threatening inflammatory disease of the intestine and is thought to promote development of new BPD as a consequence of systemic inflammation and activation of pulmonary *Tlr4*. *Tlr4* is a proinflammatory cytokine that is commonly upregulated in infants with new BPD [27,28]. Herein, we performed an immunohistochemical analysis to observe the localization of *Tlr4* expression in the lungs of F2_NONE_ and F2_TCDD_ pups. We report that maternal milk-fed F2_TCDD_ pups exhibited interalveolar *Tlr4* expression, which was heightened when pups received a supplemental formula diet. Formula supplementation among F2_NONE_ pups also led to an increase in interalveolar *Tlr4* expression when compared to their maternal milk-fed counterparts. However, after intervening with a paternal fish oil diet preconception, we found that interalveolar *Tlr4* expression was eliminated in maternal milk-fed and formula-supplemented F2_TCDD_ pups, as well as formula-supplemented F2_NONE_ pups (Figure 5A–H).

We also measured pulmonary *Tlr4* relative mRNA transcript and protein expression in all F2 pups. We found that F2_TCDD_/Form pups exhibited a significant increase in *Tlr4* mRNA expression compared to F2_None_ pups (*p* = 0.0415) while *Tlr4* protein was significantly increased in both F2_TCDD_ and F2_TCDD_/Form pups (*p* = 0.0232; *p* = 0.0149, respectively) (Figure 5). Notably, formula supplementation of pups in the absence of paternal TCDD exposure did not have a significant impact on *Tlr4* mRNA or protein expression.

### 2.3. Impact of Paternal and Neonatal Diet on Pulmonary Fibrosis in F2 Neonates

**Figure 6:**
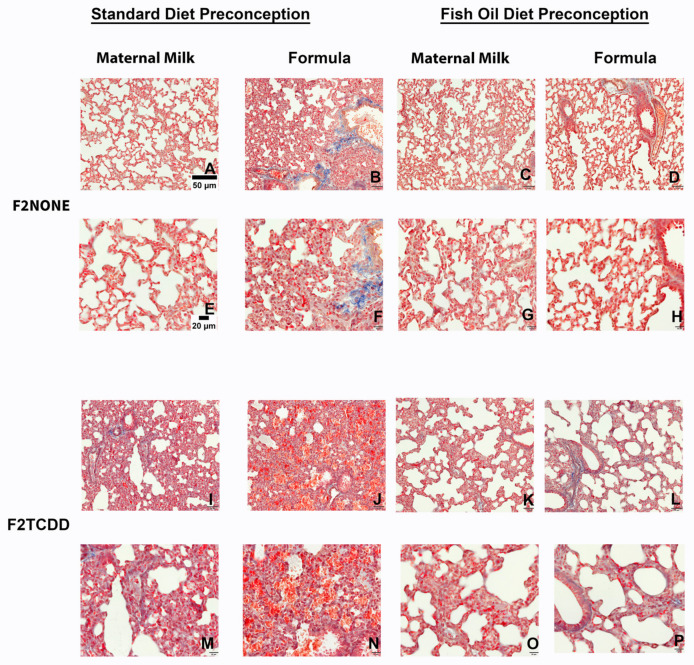
Impact of paternal and neonatal exposures and risk of pulmonary fibrosis. Representative images from multiple lungs (*N* = 3/group) of Masson’s Trichrome-stained perfused lung tissue on postnatal day 11. Original magnification 200× (**A**–**D**,**I**–**L**) and 400× (**E**–**H**,**M**–**P**). F2_NONE_ (**A**,**E**), F2_NONE_/Form (**B**,**F**), F2_NONE_/Fish (**C**,**G**), F2_NONE_/Fish/Form (**D**,**H**), F2_TCDD_ (**I**,**M**), F2_TCDD_/Form (**J**,**N**), F2_TCDD_/Fish ( **K**,**O**), F2_TCDD_/Fish/Form (**L**,**P**).

Finally, we performed Masson’s Trichrome staining to assess the incidence of pulmonary fibrosis in all F2 groups (Figure 6). Although it is normal for collagen to be deposited around pulmonary vessels [29], collagen deposition surrounding alveoli (identified by the overlap of blue and red staining) is not normal and indicates fibrosis. We found that F2_NONE_ pups displayed little-to-no signs of pulmonary fibrosis (Figure 6A,E), but collagen deposition was commonly observed in control pups receiving supplemental formula (F2_NONE_/Form pups) (Figure 6B,F). However, pulmonary fibrosis was reduced in formula-fed control pups whose fathers were provided fish oil (F2_None_/Fish/Form pups) (Figure 6D,H). F2_TCDD_ and F2_TCDD_/Form pups also demonstrated signs of pulmonary fibrosis (Figure 6I,M). However, only F2_TCDD_/Form pups exhibited interalveolar red blood cell infiltration (Figure 6J,N), consistent with our previous observations [9]. F2_TCDD_/Fish pups displayed a reduction in fibrosis and improvement in alveolarization (Figure 6K,O). Although F2_TCDD_/Fish/Form pups also exhibited improved alveolarization and their risk of interalveolar red blood cell infiltration was eliminated, pulmonary fibrosis was still apparent in approximately 50% of these pups (Figure 6L,P).

## 3. Discussion

New BPD, characterized by reduced alveolarization, most commonly occurs in premature infants and places them at increased risk of developing chronic lung disease [30]. Inflammation plays a major role in both the development and progression of new BPD and thus post-natal anti-inflammatory agents (e.g., corticosteroids) are often used therapeutically. Unfortunately, for poorly understood reasons, not all patients at risk for BPD respond to this treatment [30]. Thus, developing a better understanding of events associated with disease initiation is needed in order to identify more effective treatment or prevention strategies.

Several studies indicate that new BPD is initiated in the prenatal environment [31], suggesting to us that paternal factors capable of impeding placental function would contribute to its development. We have previously reported that, compared to control pregnancies, placentae arising in control female mice mated to TCDD exposed males exhibit reduced gene and protein expression of the progesterone receptor and insulin-like growth factor 2 as well has enhanced expression of *Tlr4* [6]. These placental changes were associated with pups being born preterm and with IUGR [6,7], conditions which are known risk factors for BPD in human infants. Indeed, using this same model, we previously reported that a paternal history of TCDD exposure was associated with an increased risk of new BPD in his offspring [10]. However, intervening with a paternal fish oil diet preconception *eliminated* the risk of PTB and IUGR [8] and significantly reduced the risk of new BPD in these pups [9]. The goal of the current study was to determine if the reduced risk of new BPD in offspring of fish oil supplemented fathers was also associated with attenuation of neonatal lung inflammation. 

Evidence from the literature suggests that hemorrhaging and edema are both associated with inflammation in BPD and other pulmonary diseases [11,32] and further compromises lung capacity [11,33]. We report here that independent of neonatal diet, F2_TCDD_ pups exhibited both lung hemorrhaging and edema denoted by gross anatomy and an increased ratio of wet-to-dry lung weight (Figure 1 and Figure 2). Our findings in mice born to toxicant-exposed fathers mirror the data of studies examining human infants with new BPD [34,35].

In the current study, we also found that intervening with a paternal fish oil diet prior to conception reduced lung hemorrhaging and edema in F2_TCDD_/Fish and F2_TCDD_/Fish/Form pups (Figure 1 and Figure 2). As stated above, both lung edema and hemorrhaging are associated with pulmonary inflammation, most likely as a consequence of pro-inflammatory mediators within alveolar interstitial fluid [36,37,38]. Therefore, we next measured pro-inflammatory mediator expression in neonatal lungs in concert with the assessment of gross morphology (hemorrhaging and edema). 

Our studies revealed that compared to all F2_none_ groups, a paternal history of TCDD exposure was associated with heightened pulmonary expression of *Cxcr2* and *Il-1 alpha* transcripts in neonatal mice. However, gene expression was normalized in pups whose fathers were provided the fish oil diet. Interestingly, formula supplementation alone had virtually no impact on *Cxcr2* or *Il-1 alpha* expression (Figure 3 and Figure 4). These findings suggest that indirect fish oil supplementation can reduce the expression of *Cxcr2* and its downstream mediator, *Il-1 alpha*. These data also support other studies that suggest the antagonism of *Cxcr2* is a potential therapeutic approach for new BPD [21]. 

We next measured *Tlr4* protein and mRNA expression since studies from others have suggested that antagonism of this receptor can protect against lung inflammation and associated diseases [39,40]. We report that, compared to control pups, *Tlr4* mRNA and protein levels were increased within the alveolar space of lungs of F2_TCDD_ pups arising from standard diet fathers. However, F2_TCDD_ pups whose fathers were provided a fish oil supplemented diet exhibited *Tlr4* levels similar to that of control pups. Unexpectedly, formula feeding of pups did not have a significant impact on *Tlr4* expression unless the paternal parent had a history of TCDD exposure. The lungs also appeared to contain interstitial fluid (Figure 5). This finding supports the work of others suggesting that infants with new BPD are at risk of developing pulmonary edema due to proinflammatory interstitial fluid within the alveolar space [41,42].

Lastly, we report that control pups provided formula (F2_none_/Form), and F2_TCDD_ pups with and without formula all exhibited signs of lung fibrosis; however, abnormal collagen deposition was not observed in F2_None_/Fish/Form and F2_TCDD_/Fish pups. Surprisingly, paternal fish oil supplementation did not reduce the risk of lung fibrosis in F2_TCDD_/Fish/Form pups despite the absence of interalveolar red blood cell infiltration (Figure 6). These results suggest that historical TCDD exposure and postnatal formula supplementation act as independent risk factors for pulmonary fibrosis. Our results also indicate that intervening with a paternal fish oil diet can reduce formula-driven lung fibrosis in the *absence* of historical TCDD exposure, but not in the presence of this variable. 

The omega-3 fatty acids are known for their anti-inflammatory effects [43,44] and supplementation with fish oil has been found to lower the risk of internal bleeding [45] and edema [46]. Overall, our data also indicate that fish oil can reduce pulmonary inflammation, as well as associated hemorrhaging, edema, and fibrosis [7,35]. However, our studies are unique in that the fish oil intervention was provided to the *paternal parent prior to conception*. Although F2_TCDD_ and F2_TCDD_/Form pups frequently exhibited new BPD, paternal fish oil supplementation attenuated the expression of pulmonary inflammation (*Cxcr2*, *Il-1 alpha*, and *Tlr4*) and significantly reduced the incidence of lung disease in offspring. As stated above, antagonism of *Cxcr2* has been suggested as a potential treatment for new BPD [21]. Our studies revealed that preconception fish oil was effective in reducing pulmonary *Cxcr2* expression in offspring and thus may have value as a *preventive* measure. Since fish oil was not provided to the F2 generation directly, this intervention likely improved lung development and health outcomes by reducing placental inflammation during pregnancy which also reduces the risk of PTB and IUGR [8]. Our studies not only provide strong evidence that new BPD can begin in utero, but that effective treatment for this devastating disease can be initiated *prior to pregnancy*.

Development of new BPD remains a major problem facing premature infants; thus, identifying effective preventative measures is a paramount concern. Our studies in mice demonstrating that the toxicant exposure and dietary history of the father can significantly impact the development of this disease may also shed light on factors that influence BPD in human infants. Due to the potential importance of our findings using a small cohort of animals, future studies will need to be conducted with a larger sample size as well as in additional experimental models of new BPD. 

## 4. Materials and Methods

### 4.1. Animals

Adult (10–12 weeks) and neonatal C57BL/6 mice were used in this study. Adult mice were obtained from Envigo (Indianapolis, IN, USA) or born in-house. All neonatal mice were born in-house. Animals were housed in the Barrier Animal Care Facility at Vanderbilt University Medical Center, which is free of common mouse pathogens. Adult mice were provided food and water ad libitum. Animal room temperatures were maintained between 22–24 °C and relative humidity of 40–50% on a 12-h light: dark schedule. Experiments described in this study were approved by Vanderbilt University’s Institutional Animal Care and Use Committee (IACUC) per the Animal Welfare Act protocol #M2000098.

### 4.2. Chemicals

Esbilac Puppy Milk Replacer Powder was purchased from Pet-Ag, Inc (Hampshire, IL, USA). All other chemicals were obtained from Sigma-Aldrich unless otherwise stated.

### 4.3. Exposure, Mating and Diet Scheme 

Virgin 10 to 12-week-old C57BL/6 females were mated with intact males of similar age. Females were weighed daily and monitored for the presence of a vaginal semen plug; denoting copulation had occurred. The morning a vaginal plug was identified, the dam was considered pregnant [denoted as embryonic day 0.5 (E0.5)] and moved to a new cage. Following confirmation of pregnancy, dams were exposed to TCDD (10 µg/kg) in corn oil or corn oil vehicle alone by gavage on E15.5 at 1100 h CST. Dams provided vehicle only were used as unexposed controls while dams receiving TCDD were designated F0 mice (the founding generation).

Although the selected dose of TCDD is higher than typical human exposures, this dose reflects the more rapid clearance of this toxicant in mice compared to humans. This dose is well below the LD50 for adult C57BL/6 mice (230 µg/kg) [47] and is not overtly teratogenic or abortogenic. In our hands, parturition typically occurs on E20 for both control and F0 pregnancies. Finally, since the half-life of TCDD is 11 days in C57BL/6 mice, offspring of F0 dams (F1 pups) are directly exposed to TCDD in utero and during lactation [47]. Germ cells present within F1 feti are also directly exposed to TCDD; these cells have the potential to become the F2 generation. 

### 4.4. Diet and Mating Scheme for the F1 Generation

Purina Mills (TestDiet division) provided the 5% Menhaden fish oil diet, which also contained 1.5% corn oil to prevent depletion of omega-6 fatty acids. Menhaden fish oil, (OmegaProtein, Houston, TX, USA), has an established fatty acid profile (~40% omega-3 fatty acids) and was processed to remove dioxins and polychlorinated biphenyls. The fish oil diet is a modification of Purina’s low phytoestrogen rodent chow, 5VR5, which was used as the control (standard) diet. Protein, total fat, and energy content are similar across diets. The fish oil diet was maintained in vacuum-sealed bags at −20 °C until use and once provided to mice, replaced every 3 days.

After weaning, F1 males were maintained on a standard or fish oil diet for 7-weeks (one full cycle of spermatogenesis) and mated at 10–12 weeks of age with age-matched unexposed C57BL/6 females. Once a vaginal semen plug was identified, dams were singly housed until parturition. 

### 4.5. Formula Feeding

Beginning on postnatal day 7 (PND7), pups were weighed, and randomized to a strict maternal milk diet or a supplemental formula diet. Pups were bottle-fed 30 µL of formula three times a day over the course of four days using a small nipple attached to a 1ml syringe (Miracle Nipple Mini for Pets and Wildlife). Each 30 µL dose was provided in two aliquots of 15 µL, 10 min apart. All pups (independent of neonatal diet) remained with dams for the duration of the study and formula-supplemented pups were allowed to nurse ad libitum. Pup nomenclature is described in Table 1. 

### 4.6. Euthanasia and Sample Collection

On PND11 at 1100 h local time, pups were weighed, then euthanized by decapitation performed under deep anesthesia per AAALAC guidelines. Following euthanasia, the rib cavity was opened, and the lungs were identified. The lungs were weighed, and some were dried to obtain the dry weight or stored at −80 °C until further use.

### 4.7. qRT-PCR

Lung tissue was lysed using the Trizol reagent (Invitrogen, Carlsbad, CA, USA) and total RNA was purified from tissue lysates using the RNeasy Mini Kit (Qiagen, Valencia, CA, USA). RNA quality was verified using Nanodrop and RNA with a 260/280 ratio of ~2.0 were used for qRT-PCR. An iScript cDNA synthesis kit (Bio-Rad, Hercules, CA, USA) was used to generate cDNA from 1 µg of total RNA using random decamer primers as described by the manufacturers. The same thermal cycling program was applied *Cxcr2*, *Il-1 alpha* and *Tlr4*: 95 °C for 30 s, 40 cycles of 95 °C for 5 s, 60 °C for 5 s using a Bio-Rad CFX96 Real-time thermocycler. The melt curve was analyzed to confirm product purity. All reactions were performed in triplicate. *Ribosomal 18s* transcript was used as a housekeeping gene to normalize transcript levels of *Cxcr2*, *Il-1 alpha*, and *Tlr4* (Sino Biological, Chesterbrook, PA, USA) for all samples (primer sequences were not disclosed by the company). Results were evaluated using the delta-delta Ct method as previously described [9].

### 4.8. Masson’s Trichrome Stain

At the time of necropsy, the whole lung tissue from a subset F2 mice was perfused with phosphate-buffered saline (PBS), embedded in paraffin and sectioned at 5 µm as previously described [9]. Lung slides were deparaffinized in xylene, then rehydrated in increasing concentrations of ethanol, followed by dH2O. Slides were then incubated overnight at room temperature in Bouin’s solution. The next day, slides were washed and incubated with Wiegert’s iron hematoxylin solution for 10 min, then washed. Next, slides were stained with Biebrich scarlet-acid fuchsin solution for 15 min then washed. Slides were then differentiated by incubating in phosphomolybdic- phosphotungstic acid solution for 20 min. Without washing, aniline blue solution was added, and the slides incubated for an additional 15 min. Slides were then washed briefly and differentiated by incubating in a 1% acetic acid solution for 3 min. Slides were then washed and dehydrated in increasing concentrations of ethanol and xylene. Slides were coverslipped and viewed using an Olympus B071 microscope. Collagen is denoted by blue staining; nuclei are denoted by black staining and muscle/cytoplasm/keratin are denoted by red staining. 

### 4.9. Lung Hemorrhaging and Edema Analysis

At the time of necropsy the whole lung was quickly removed from the chest cavity and immediately photographed. This photograph was used to assess lung hemorrhaging via gross analyses (e.g., coagulation of blood at the surface of the lung or discoloration) as previously described [48,49]. Next, the right lobe of the lung was excised from the whole lung and weighed. The right lobe was then placed in an oven set at 20 °C for 5 days. On the 6th day, the lung lobes were reweighed. The wet lung weight was then divided by the dry lung weight and an increased ratio was a sign of edema. Herein, a ratio of 5 or greater was classified as edema and a ratio of less than 5 suggested no edema as previously described [50,51]. 

### 4.10. Immunohistochemistry

Immunohistochemistry: At the time of necropsy, the whole lung tissue from some F2 mice was perfused as previously described [3]. Slides were deparaffinized in xylene, then rehydrated in increasing concentrations of ethanol, followed by dH_2_O. Antigen retrieval was performed by placing slides in citrate buffer within a warm rice cooker. After a dH2O wash, endogenous peroxidase activity was blocked by incubating slides in 3% hydrogen peroxide in methanol. Slides were washed in phosphate buffered saline (PBS) and blocked for non-specific binding using powerblock reagent (BioGenex, Freemont, California; Cat# HK085-5K). Slides were washed in PBS and incubated overnight with rabbit Toll like 4 Receptor Primary antibodies (Abcam, Boston, MA, USA; Cat# ab13556) diluted 1:200 in PBS containing 0.5% triton x-100 (PBST) in a humidifying chamber. After a PBST wash, slides were incubated with a premade goat anti-rabbit secondary antibody (Abcam, Waltham, Massachusetts; Cat# ab64256) for 20 min at room temperature. After a PBST wash, slides were incubated with streptavidin peroxidase (Thermo Scientific, Waltham, MA, USA; Cat# TS-125-HR) for 20 min at room temperature. After a PBS wash, color was developed using the Vector DAB peroxidase chromogen kit (Vector Laboratories, Newark, CA, USA; Cat# ZG0306) as described by the manufacturer. After washing in dH20, slides were counterstained with Hematoxylin (Ricca, Arlington, TX, USA; Cat# 3537-32). Next, slides were placed under warm running water for a bluing effect, then dehydrated in increasing concentrations of ethanol and xylene. Slides were coverslipped and viewed using an Olympus B071 microscope. DAB was quantified using the DAB quantification Image J plugin.

### 4.11. Statistics

All data were analyzed using GraphPad Prism’s one-way ANOVA and the Tukey post-hoc test. For all experiments, three to six non-littermates were used to obtain the average for each group. The presented images are representative of each group. The data are represented as the mean ± standard deviation. Values of *p* < 0.05 were considered significant. Significance was determined by comparing each treatment group to Control (CT)/F2_NONE_ pups. All experiments were repeated twice using different non-littermates. In each group, approximately half of the pups were male, and the other half were female. The majority of the pups were male in groups with uneven samples sizes.

## Figures and Tables

**Table 1:** Description of pup nomenclature used throughout the manuscript.

F2 Generation Nomenclature	Was the Pup’s Father (F1 Generation) Exposed to TCDD in Utero?	Did the Pup’s Father (F1 Generation) Receive a Fish Oil Preconception Diet?	Did the Pup (F2 Generation) Receive Postnatal Formula Supplementation?
F2_NONE_ (CT)	No	No	No
F2_NONE_/Fish (CT)	No	Yes	No
F2_NONE_/Form (CT)	No	No	Yes
F2 _NONE_/ Fish/Form (CT)	No	Yes	Yes
F2_TCDD_	Yes	No	No
F2_TCDD_/Fish	Yes	Yes	No
F2_TCDD_/Form	Yes	No	Yes
F2_TCDD_/Fish/Form	Yes	Yes	Yes

“CT” represents “control” groups that do not have a history of TCDD exposure.

## Data Availability

Not applicable.

## Supplementary Materials

The following supporting information can be downloaded at: https://www.mdpi.com/article/10.3390/md21030161/s1, Figure S1: Wet lung weights; Figure S2: Dry lung weights.
