# Supplementary figures and images for: A Paternal Fish Oil Diet Preconception Reduces Lung Inflammation in a Toxicant-Driven Murine Model of New Bronchopulmonary Dysplasia
Source: Mar Drugs. 2023 Feb 27;21(3):161. doi: 10.3390/md21030161 (PMC10052688; doi:10.3390/md21030161)

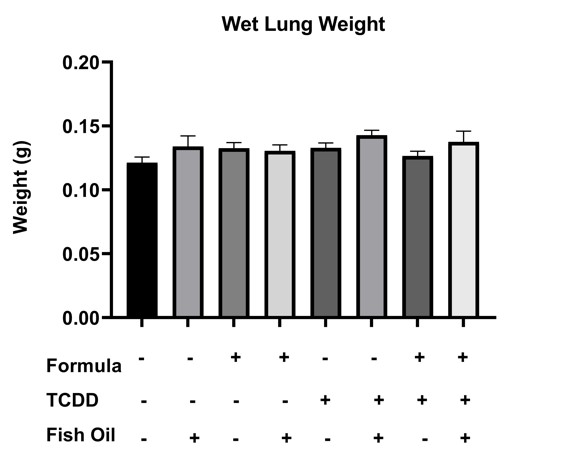

Supplement: Supplementary file 1 [file marinedrugs-21-00161-s001.zip › Figure S1.jpg]

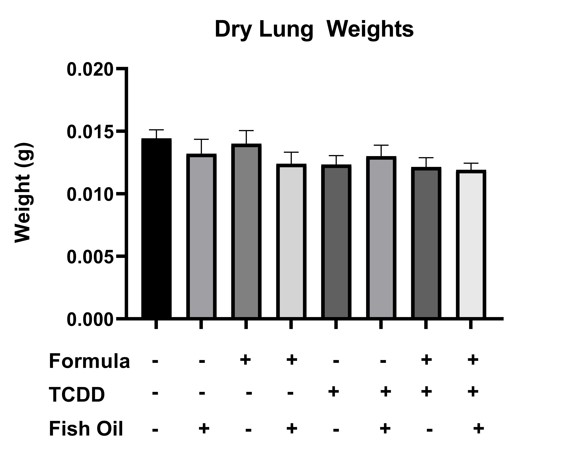

Supplement: Supplementary file 1 [file marinedrugs-21-00161-s001.zip › Figure S2.jpg]
